# Mitophagy–NLRP3 Inflammasome Crosstalk in Parkinson’s Disease: Pathogenic Mechanisms and Emerging Therapeutic Strategies

**DOI:** 10.3390/ijms27010486

**Published:** 2026-01-03

**Authors:** Sahabuddin Ahmed, Tulasi Pasam, Farzana Afreen

**Affiliations:** 1 Department of Psychiatry, Yale University School of Medicine, New Haven, CT 06510, USA; tulasi.pasam@yale.edu; 2 Independent Researcher, New Haven, CT 06511, USA; farzanaafreen59@gmail.com

**Keywords:** Parkinson’s disease, NLRP3 inflammasomes, mitophagy, PINK1/Parkin pathway, neuroinflammation, neuroprotection

## Abstract

Parkinson’s disease (PD) is a progressive neurodegenerative disorder characterized by the loss of dopaminergic neurons in the substantia nigra and pathological α-synuclein aggregation. Growing evidence identifies chronic neuroinflammation—particularly NLRP3 inflammasome activation in microglia—as a central driver for PD onset and progression. Misfolded α-synuclein, mitochondrial dysfunction, and environmental toxins act as endogenous danger signals that prime and activate NLRP3 inflammasome, leading to caspase-1–mediated maturation of IL-1β and IL-18 and subsequent pyroptotic cell death. Impaired mitophagy, due to defects in PINK1/Parkin pathways or receptor-mediated mechanisms, permits accumulation of dysfunctional mitochondria and release DAMPs, thereby amplifying NLRP3 activity. Studies demonstrate that promoting mitophagy or directly inhibiting NLRP3 attenuates neuroinflammation and protects dopaminergic neurons in PD models. Autophagy-inducing compounds, along with NLRP3 inhibitors, demonstrate neuroprotective potential, though their clinical translation remains limited due to poor blood–brain barrier penetration, off-target effects, and insufficient clinical data. Additionally, the context-dependent nature of mitophagy underscores the need for precise therapeutic modulation. This review summarizes current understanding of inflammasome–mitophagy crosstalk in PD, highlights major pharmacological strategies under investigation, and outlines its limitations. Future progress requires development of specific modulators, targeted delivery systems, and robust biomarkers of mitochondrial dynamics and inflammasome activity for slowing PD progression.

## 1. Introduction

Parkinson’s disease (PD) is a slowly progressive neurological disorder that affects approximately 1% of individuals aged >65 years, increasing to 4–5% among those aged 85–90 years [1,2]. Clinically, PD is characterized by resting tremor, bradykinesia, rigidity and postural instability, primarily due to the degeneration of dopaminergic neurons in the substantia nigra par compacta (SNpc). This neuronal loss leads to dopamine depletion in the caudate–putamen, disrupting basal ganglia circuity responsible for motor coordination [3,4]. In addition to motor impairments, PD patients exhibit a wide range of non-motor symptoms including sleep disturbances, depression, and cognitive decline. Dementia is a major complication affecting 47% of PD patients, and its prevalence substantially increase with disease progression [5]. The chief pathological hallmark of PD is the presence of Lewy bodies and intraneuronal cytoplasmic composed of insoluble α-synuclein aggregates [6,7]. The accumulation of misfolded and fibrillar α-synuclein fibrils in dopaminergic neurons contributes to neuronal dysfunction and degeneration [8]. Several mechanisms have been proposed to underlie PD pathogenesis, including chronic neuroinflammation, mitochondrial dysfunction, oxidative stress, defective autophagic clearance of misfolded proteins and damaged organelles [9,10].

The immune cell of the central nervous system (CNS) includes microglia, astrocytes and dendritic cells, which collectively maintain neuronal surveillance and regulate extracellular signaling to maintain brain homeostasis [11,12,13]. Microglia are the principal immune cells of the brain, displaying a highly ramified morphology, a small cytoplasmic volume, a polygonal nucleus, and abundant rough endoplasmic reticulum (RER) in the resting stage [14,15]. Neuroinflammation in the PD brain is closely associated with microglial activation and occurs in parallel with dopaminergic neurons death [16]. Activated microglia, positive for human leukocyte antigen-D related (HLA-DR) in the SNpc and putamen of post-mortem PD brains, were first reported by Mc Geer et al. providing evidence of neuroinflammation in PD pathogenesis [17,18]. Microglial activation may result from chronic infections, repeated tissue injury, or accumulation of misfolded proteins, which act as stress signals or damage-associated molecular patterns (DAMPS) [19,20]. While transient microglial activation is beneficial for clearing debris and pathogens, sustained or excessive activation leads to overproduction of pro-inflammatory mediators, including cytokines, chemokines and reactive oxygen species (ROS), ultimately causing neuronal damage and neurodegeneration [21,22]. Beside maintaining neuronal homeostasis by purging foreign particles and response to tissue damage or bacterial endotoxin, microglia are also involved in tissue remodeling and adaptation via continuous surveillance [23,24]. Microglia express both immune- and neuron-derived receptors on their surface and thus regulate immune as well as neuronal activity [24,25]. Furthermore, microglia establish dynamic contacts with neurons and executes several physiological functions, including synaptic plasticity, neuronal differentiation and production of neurotropic factors during brain development [26,27]. Thus, long-term distortion of microglia activation, shifting from a resting to a chronically active state, can lead to serious neurological and psychiatric disorders, as well as neurodegenerative diseases, including PD, Alzheimer’s disease, Huntington disease, and more [28,29]. The generation and maturation of inflammatory cytokines, such as interleukin-1β (IL-1β) and interleukin-18 (IL-18), involve activation of the intracellular multimeric protein complex known as NLRP3 inflammasomes, which acts as sensors for pathogen-associated molecular patterns (PAMPs) or DAMPs and contribute to the development of autoimmune and neurodegenerative disorders [29]. Therefore, regulation of NLRP3 inflammasome activation represents a promising therapeutic strategy for PD.

Autophagy is a highly evolved catabolic process in eukaryotes that mediates the elimination and degradation of misfolded proteins and dysfunctional or redundant organelles. Factors such as nutrient deprivation, energy deficiency, and accumulation of misfolded proteins induce autophagy, leading to lysosomal degradation and generation of recycle metabolites that can be reused in several biosynthetic pathways [30,31]. One of the major pathways through which α-synucleins are degraded and lysed is through autophagy [30,32]. Accumulating evidence supports the involvement of aberrant autophagy in PD pathogenesis [33,34]. Neurotoxin such as 1-methyl-4-phenyl-1,2,3,6-tetrahydropyridine (MPTP) or rotenone, which inhibit mitochondrial complex I, lead to mitochondria dysfunctional and impaired autophagic flux, resulting in neuronal death [35,36]. Hence, timely removal of these misfolded protein or damaged mitochondria by stimulating the autophagy machinery by a pharmacological agent has emerged as a potential therapeutic strategy for neurodegenerative disorders particularly PD [37].

Several regulatory mechanisms suppress NLRP3 inflammasome activation, among which autophagy, especially mitophagy, has emerged as critical modulator. Induction of mitophagy eliminates damaged mitochondria and endogenous dangerous signals, thereby preventing excessive inflammasome activation [37]. Conversely, impaired mitophagy can augment NLRP3, highlighting their inverse regulatory relationship; however, the precise molecular mechanism is yet to be defined [38].

While earlier reviews have discussed mitophagy or NLRP3 inflammasome activation independently, a significant knowledge gap remains regarding how their bidirectional crosstalk influences PD pathogenesis. Findings from the last decade demonstrating how defective mitochondrial turnover exacerbates neuroinflammation are rarely integrated, and the current literature does not thoroughly evaluate therapeutic strategies targeting both pathways simultaneously. To address this gap, the present review is based on a comprehensive and critical evaluation of peer-reviewed studies retrieved primarily from PubMed, Web of Science, and Scopus databases using combinations of keywords including “Parkinson’s disease,” “NLRP3 inflammasome,” “mitophagy,” “autophagy,” “neuroinflammation,” “microglia,” and “mitochondrial dysfunction.” The review focuses mainly on studies published in English between 2015 and 2025, with inclusion of seminal earlier works where necessary to provide historical and mechanistic context.

This review differs from the existing literature by providing an updated and integrated perspective on how disrupted mitophagy and hyperactive NLRP3 signaling jointly drive PD progression rather than offering a broad overview of all PD-related pathways. Studies were included if they directly examined NLRP3 inflammasome signaling, mitophagy or autophagy processes, or mechanistic interactions between these pathways in PD or PD-relevant experimental models. In contrast, studies were excluded if they focused exclusively on unrelated inflammatory pathways, lacked relevance to inflammasome activation or mitochondrial quality-control mechanisms, or did not provide mechanistic insight into neurodegeneration

## 2. Inflammasomes Expression, Activation and Regulation

### 2.1. Inflammatory Mechanisms in the Brain

Inflammation in the brain is an innate immune response triggered by noxious stimuli that may threaten cellular homeostasis. This response requires the immediate elimination of harmful agents and the promotion of tissue repair [39]. However, persistent low-grade neuroinflammation disrupts neuronal homeostasis and activates various pro-inflammatory mediators, especially IL-1β, IL-18, caspase-1, and IL-33, all of which can contribute to neurodegeneration [40]. These cytokines are widely studied because of their established roles in neuronal deficits, which make them emerging therapeutic targets.

Accumulating preclinical and clinical evidence indicates that IL-1β and caspase-1 play a pivotal role in the ignition and propagation of neuroinflammation in PD [41]. These mediators are processed and activated by the inflammasomes’ complex and multimeric immune sensors whose involvement has been established in several neurodegenerative disorders, including PD [11]. Once activated, they promote caspase-1-mediated cleavage of pro-IL-1β and pro-IL-18 into their active form [42,43]. The maturation and release of active IL-1β and IL-18 further amplify inflammatory cytokines production, increase immune cell infiltration, and induce pyroptotic cell death in both neuron and glial populations [44,45].

Previous studies have consistently demonstrated that chronic IL-1β signaling accelerates dopaminergic neuronal loss in both toxin-induced and genetic PD models (e.g., MPTP, rotenone models), supporting its direct pathogenic contribution [46]. Likewise, elevated caspase-1 activity has been reported in post-mortem PD substantia nigra tissue, reinforcing its clinical relevance [47]. Caspase-1-dependent pyroptosis also represent a link between inflammation and programmed cell death, further justifying a focused discussion on inflammasome signaling in context of PD pathogenesis.

However, it is important to critically evaluate whether elevated IL-1β, IL-18, or caspase-1 levels in cerebrospinal fluid truly reflect NLRP3-dependent neuroinflammation in PD, as these cytokines can also be produced through inflammasome-independent mechanisms and may represent broader systemic or neuroinflammatory states rather than specific NLRP3 activation [48,49]. Therefore, while their elevation supports inflammatory involvement, it should not be interpreted as a definitive surrogate for NLRP3 activity without complementary mechanistic evidence. Together, these findings provide strong evidence that sustained inflammasome-mediated cytokine production is not merely a secondary response but a major driver of PD-associated neurodegeneration.

While oxidative stress, apoptosis, mitochondrial dysfunction, and protein aggregation are well-recognized contributors to the pathogenesis of PD and cannot be overlooked, accumulating evidence indicates that neuroinflammation is not merely a downstream consequence of neuronal injury. Rather, inflammatory signaling can act as an early driver that can precede and amplify oxidative stress and apoptotic pathways. In this context, inflammasome mediators, particularly NLRP3, have emerged as key mediators that integrate multiple pathogenic signals, including mitochondrial dysfunction, ROS overproduction, lysosomal damage, and misfolded α-synuclein. Therefore, this review focuses specifically on NLRP3 inflammasome signaling and highlights upstream regulatory processes, such as mitophagy, to provide a framework for early disease mechanisms and to improve understanding of PD pathogenesis.

### 2.2. Inflammasome Complex: Structure and Function

Whenever an immune cell senses any dangerous stimuli in the form of PAMPs or DAMPs, inflammasomes assemble in the cytoplasm, become activated, and arbitrate consequent immune responses. Several inflammasome sensors have been identified, including members of the nucleotide-binding domain leucine-rich repeat (NLR) family—NLRP1, NLRP2, NLRP3, and NLRC4—as well as PYHIN family members (non-NLR family), such as absent in melanoma 2 (AIM2) and IFI16 [50,51]. All of them can sense dangerous signals and become activated to form the human immune defense system [51]. Most inflammasomes share a common tripartite architecture consisting of an inflammasome sensor molecule, the adaptor protein ASC (apoptosis-associated speck-like protein) containing a caspase 1 activation and recruitment domain (CARD) as well as the effector protease caspase-1 [52,53]. Interactions between these components induce conformational changes that activate immune activity and are therefore tightly regulated.

Several studies have demonstrated that, among the known inflammasome sensors, NLRP3 is the most strongly implicated in PD pathology. Prior work shows that mitochondrial ROS, α-synuclein fibrils, and lysosomal damage—key pathological features of PD—directly trigger NLRP3 activation in microglia [54]. In vivo studies further support this, as NLRP3 knockout mice exhibit reduced neuroinflammation and are partially protected from dopaminergic neuron loss in MPTP models [55]. Additionally, clinical studies report elevated NLRP3 components in the serum and CSF of PD patients [56], underscoring its potential translational relevance.

### 2.3. Structure and Activation Mechanism of NLRP3 Inflammasome

Among known inflammasomes, NLRP3 is the most extensively studied in neurodegenerative disorders and is therefore considered the prime inflammasome discussed in this review [57]. Early studies suggested that NLRP3 activation required bacterial pathogens or extracellular ATP; however, recent work has demonstrated that a wide array of stimuli and environmental insults—including’s DAMPS, metabolic stress, and particulate matters—can activate NLRP3 [58,59]. Like other inflammasomes, NLRP3 also consists of a NOD-like receptor (NLR3) domain, characterized by three distinctive regions: a central NACHT domain, a leucine-rich repeats (LRRs) domain, and a PYD or CARD mediating ASC/caspase-1 activation. These domains coordinate recognition of stimulus and ensure that NLRP3 activation occurs only under appropriate conditions, making the process highly regulated [42,60].

Activation induces oligomerization of the NLRP3 inflammasomes in brain microglia; it is governed by both transcriptional and post-translational mechanisms that require two check points, priming/transcription (Signal 1) and activation/assembly (Signal 2) [61]. The priming step is mediated by Toll-like receptor (TLR)-adaptors, including pathogenic stimuli (e.g., lipopolysaccharide), tumor necrosis factor receptor (TNF) receptors, or IL-1 receptors. This step does not directly activate the NLRP3 inflammasome but primes it for activation. Priming involves activation of the transcription factor NF-κB, which translocates to the nucleus and increases transcription of key inflammasomes-related genes, including NLRP3, IL-1β, and IL-18 [62,63]. However, controversy remains regarding the exact mechanisms by which TLR4-mediated NF-κB activation primes NLRP3 [59]. Some studies reported that inhibiting NF-κB does not completely prevent NLRP3 activation [64]. Other studies demonstrate that NLRP3 can also be regulated at the post-translational level, as simultaneous exposure to Signal 1 and Signal 2, for as little as 10 min, is sufficient to enhance inflammasome activation [65]. Juliana et al. showed that non-transcriptional priming via Signal 1 in mouse macrophage regulates activation of NLRP3 deubiquitnation, even without transcriptional changes [66]. Thus, further investigation is required to elucidate the precise regulatory mechanism underpinning the regulation of NLRP3.

**Figure 1:**
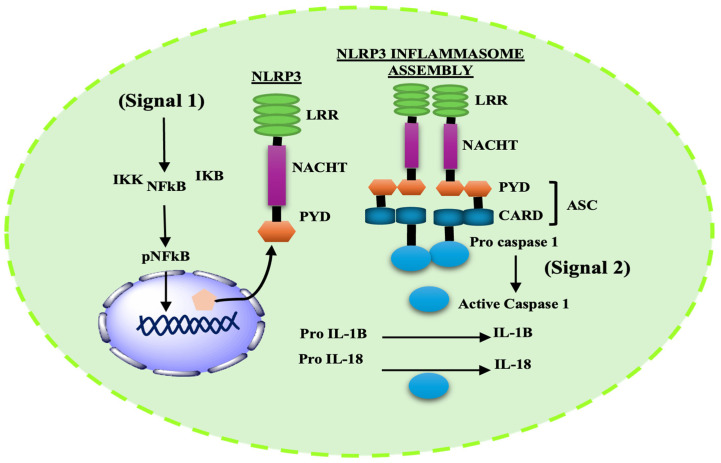
Two-step signaling cascade for NLRP3 inflammasome activation. Signal 1 (Priming): TLR4 stimulation activates NF-κB, leading to transcriptional upregulation of NLRP3, pro–IL-1β, and pro–IL-18. Signal 2 (Activation) (e.g., mitochondrial stress, K^+^ efflux, ROS, or lysosomal damage) promotes NLRP3 oligomerization and assembly with ASC and pro–caspase-1, resulting in caspase-1 activation and maturation of IL-1β and IL-18.

Activation (Signal 2) is triggered by PAMPs, DAMPs, or toxins. The NLRP3 PYD recruits ASC through PYD–PYD interactions, while the CARD of ASC recruits pro-caspase 1 through CARD–CARD interaction and subsequently activates the complex. This arrangement triggers the autocatalytic cleavage of the zymogen pro-caspase 1 into its active caspase-1 p20/p10 [67,68] (Figure 1).

## 3. NLRP3 Inflammasomes in Parkinson’s Disease

PD is a progressive neurodegenerative disorder characterized by dopaminergic neuron loss in the SNpc and accumulation of α-synuclein aggregates [69]. Neuroinflammation appears early in PD pathology and progress alongside disease severity. Misfolded α-synuclein acts as an endogenous DAMP that activates through the NLRP3 inflammasomes [70]. Both monomeric and fibrillar α-synuclein can induce pro-IL-1β expression via TLR2 signaling, but fibrillar α-synuclein is the primary trigger for NLRP3 activation, largely through mechanisms involving phagocytosis and cathepsin B release [71,72]. IL-1β is one of the potent pro-inflammatory cytokines and promotes a wide range of immunological processes, including infection, inflammation, and autoimmunity. Elevated IL-1β levels have been verified in the nigrostriatal regions, cerebrospinal fluids, and animal models of PD [25,73]. IL-18 is another cytokine whose role has been established in several neurological disorders, including AD, schizophrenia, multiple sclerosis, and depression [74,75]. The generation of both IL-1β and IL-18 is synchronized by caspase-1, a key mediator of neuroinflammation in the PD brain.

Multiple studies in mouse models of PD demonstrate that IL-1β production is driven by NLRP3 activation in brain microglia [76]. Genetic or pharmacological inhibition of NLRP3 confers protection against neurodegeneration in PD models. A recent study using HEK293 cells identified a genetic variant of NLRP3 multiple single-nucleotide polymorphisms (SNPs), including rs7525979, which are associated with reduced PD [77]. Chronic IL-1β overexpression in the substantial nigra of rat brain exacerbates PD-like pathology, supporting a causal role of inflammasome signaling [78]. α-Synuclein pathology is also linked to caspase-1, which cleave α-synuclein and promotes aggregation. Inhibition of caspase-1, either by genetic knockdown or chemical inhibitors, reduces α-synuclein truncation and protects neurons [79]. Mice lacking caspase-1 exhibit delayed disease progression and resistance to nigral neuronal damage in the MPTP model [80]. Furthermore, caspase-1 knockout mice showed reduced MPTP- induced caspase-7 cleavage, preventing nuclear translocation of poly (ADP-ribose) polymerase 1 (PARP1), whereas over-expressing caspase-7 diminishes the protective effect of caspase-1 inhibition [80].

Emerging evidence also highlights the role of post-transcriptional regulation by microRNAs (miRs) in the regulation of genes responsible for inflammation [81]. Specifically, microRNA-30e (miR-30e) is significantly downregulated in MPTP models, and enhancement of its activity using a miR-30e agomir improves behavior outcomes and attenuate neuronal loss via NLRP3 inhibition [82]. These findings suggest novel therapeutic strategies for modulating NLRP3 activity in neurodegenerative disorders.

Mitochondrial toxins such as MPTP and rotenone are used to model PD in animals and cell lines, implicating a potential role of mitochondrial dysfunction in disease pathogenesis [83]. In MPTP-treated mice, NLRP3 deficiency confers resistance to dopaminergic neuronal loss, thus providing a strong link between NLRP3, mitochondrial dysfunction, and PD [43]. Dopamine has been shown to negatively regulate NLRP3 activation via binding to the dopamine D1 receptor (DRD1), promoting NLRP3 ubiquitination and degradation via E3ubiquitin ligase. Additionally, deletion of DRD1 in MPTP-treated mice exacerbates dopaminergic neuronal damage due to enhanced NLRP3 expression [84]. Dopamine D2 receptor signaling in astrocytes similarly inhibits NLRP3 activation through β-arrestin-2 pathways [85]. Collectively, these findings indicate a bidirectional regulatory relationship between dopamine and NLRP3 inflammasome activity. Rotenone, a mitochondrial complex I inhibitor and environmental risk factor for PD, induces bioenergetic deficits that intensify NLRP3 signaling and promote dopaminergic neuronal death [11,86]. Rotenone treated wild-type mice exhibit increased serum cytokine levels, which was not observed in NLRP3 knockout mice. Similarly, brain samples of mice treated with rotenone showed NLRP3-dependent inflammation and nigral damage, while NLRP3 knockout mice showed neuroprotection [41,87]. Collectively, these findings identify NLRP3 as a key mediator of dopaminergic neurodegeneration driven by α-synuclein pathology, mitochondrial toxins, and inflammatory stress, supporting its relevance as a therapeutic target in PD.

However, it is important to note the limitations of the MPTP and rotenone models. Although these models robustly reproduce mitochondrial dysfunction and dopaminergic neurotoxicity, they do not fully capture the complex, multifactorial nature of sporadic PD, including progressive α-synuclein aggregation, widespread non-dopaminergic pathology, or long-term disease evolution [88,89]. Moreover, their acute or subacute toxin-based mechanisms may not accurately reflect the chronic, idiopathic origins of PD in humans.

In addition, data from human tissues and human-induced pluripotent stem cell (iPSC)-derived neuronal models remain comparatively limited. Incorporating findings from post-mortem human PD brains and human iPSC-based systems—particularly those modeling patient-specific genetics, mitochondrial defects, and inflammatory responses—would significantly enhance the translational relevance of NLRP3-related mechanisms and better bridge the gap between experimental models and human disease pathology [90,91]. Furthermore, the practical utility of proposed biomarkers such as circulating mitochondrial DNA (mtDNA) requires more cautious interpretation. While increased plasma mtDNA is often suggested as an indicator of impaired mitophagy or mitochondrial damage in PD, elevated mtDNA can also arise from non-specific systemic cellular stress, inflammation, or peripheral tissue injury [92]. Thus, mtDNA levels alone may not reliably distinguish PD-related mitophagy defects from generalized stress responses, underscoring the need for more specific and context-validated biomarkers.

## 4. General View of Autophagy and Its Activation

Autophagy is an evolutionarily preserved intracellular catabolic process through which cytosolic cargo, including aged or damaged organelles and aggregated proteins, are degraded via the lysosome-targeted pathways [93]. This system enables the recycling of cellular constituents to withstand metabolism under stress conditions such as nutrient deprivation, organelle deterioration, or pathogen infection, thereby preserving cellular homeostasis [30,94]. Morphologically, autophagy is characterized by the formation of bi-membraned vesicles, termed autophagosomes, which sequester cytoplasmic cargo and subsequently fuse with lysosomes (in mammalian cells) or vacuoles (in fungi). Within lysosomes, acidic hydrolases degrade the autophagic bodies, and release metabolites such as amino acids, sugars, and lipids back into the cytoplasm for reuse [95].

Autophagy comprises three main major types: macroautophagy, microautophagy, and chaperone-mediated autophagy (CMA). Macroautophagy, most often referred to as autophagy, involves autophagosomes formation, whereas macroautophagy involves the direct engulfment of cytosolic constituents by lysosomes or endosomes. CMA, different from the other two types, specifically targets proteins containing KFERQ-like motif, which are recognized by the cytosolic chaperone Hsc70 and translocated into lysosomes via the LAMP2A receptor for degradation [96]. The autophagic process consists of a series of molecular stages beginning with the formation of the phagophore (pre-autophagosomal structure), which derives membranes from multiple cellular organelles such as the endoplasmic reticulum, Golgi, endosomes, and cytosolic membrane, although the precise origin in mammalian cells remains debated [97]. The phagophore expands into a double-membraned autophagosome, which subsequently fuses with lysosomes to form autolysosomes where cargo degradation occurs [98].

Autophagy initiation is regulated by the ULK1 complex (ULK1, ULK2, ATG13, FIP200, ATG101), whose activity is suppressed by mTORC1 under nutrient-rich conditions. Vesicle nucleation is mediated by the class III PI3K complex (VPS34/PIK3C3, ATG14, UVRAG/p63, AMBRA1, Beclin1), which is negatively regulated by BCL-1 and BCL-xL through interaction with Beclin1′s BH3 domain [99,100]. Vesicle elongation involves two ubiquitin-like conjugation systems: ATG7 and ATG10 mediate ATG5–ATG12 conjugation to form a complex with ATG16L, while ATG7 also facilitates the lipidation of LC3-I to LC3-II, a process catalyzed by ATG4, ATG7, and ATG10 [101]. In selective autophagy, adaptor protein such as p62/SQSTM1 link ubiquitinated cargo to LC3-II on the phagophore membrane. Fusion of mature autophagosomes with lysosomes is mediated by the SNARE protein syntaxin-17 (STX17), enabling cargo degradation and the recycling of metabolites back into the cytoplasm [102,103].

Among autophagy subtypes, mitophagy—the selective clearance of damaged or dysfunctional mitochondria—is particularly important in maintaining mitochondrial quality control and neuronal health [104]. In PD, mitochondrial dysfunction appears as an early and persistent pathological hallmark, and impaired mitophagy disrupts mitochondrial dynamics, leading to the accumulation of defective mitochondria [54]. This accumulation increases oxidative stress, thereby directly affecting neuronal vulnerability and contributing to progressive neurodegeneration. Importantly, damaged mitochondria that fails to be efficiently cleared release mitochondrial DAMPs such as ROS, providing a mechanistic bridge between impaired mitophagy and aberrant activation of the NLRP3 inflammasomes in PD [105].

## 5. Mitophagy-Mediated Control of Mitochondrial Homeostasis and NLRP3 Inflammasomes Regulation

Both mitophagy and the NLRP3 inflammasome, rather than acting independently, cross-regulate each other in a context and time dependent manner, either protecting neurons or terminating inflammasome signaling. In PD, this bidirectional regulation becomes more relevant, as excessive inflammasome activation and impaired mitophagy synergistically contribute to neuroinflammation and dopaminergic neuron loss.

### 5.1. Mitophagy

Mitochondria are highly dynamic organelles that play a central role in maintaining cytoplasmic homeostasis. They produce essential biosynthetic intermediates and serve as key regulators of cellular metabolism, autophagy, and apoptosis [106]. Various mitochondrial complex I inhibitors, endogenous danger signals, and environmental stressors can induces excessive ROS generation, leading to mtDNA dysfunction, which has been shown to positively regulate NLRP3 inflammasome activation [43,107]. Mitophagy, a selective form of autophagy, mediates the removal of damaged mitochondria, thereby reducing ROS accumulation and limiting the release of mitochondrial DAMPs that triggers NLRP3 inflammasome activation [108]. Thus, efficient mitophagy serves as a checkpoint for inflammasome signaling.

Importantly, impaired mitophagy leads to accumulation of mitochondrial DAMPs such as oxidized mtDNA and cardiolipin, which accumulate in the cytosol and directly activate the NLRP3 inflammasome cascade [109]. Oxidized mtDNA binds the NLRP3, NACHT, and LRR domains to stabilize inflammasome assembly, while cardiolipin translocate to the outer mitochondrial membrane under stress and promotes NLRP3 recruitment and oligomerization [110]. Failure to clear these signals sustains NLRP3 activation, enhances IL-1β and IL-18 maturation, and perpetuates neuroinflammatory signaling.

#### 5.1.1. PINK1 and Parkin-Dependent Mitophagy

A diverse array of molecular mechanisms mediates mitophagy, among which the PINK1/Parkin-dependent pathway is the most extensively studied [111]. This post-translational signaling cascade selectively identifies and eliminates damaged mitochondria via a ubiquitin-dependent degradation route [112]. Under stressful conditions, mitochondrial depolarize, leading to accumulation of PINK1 on the outer mitochondrial membrane (OMM) which in turn activates the cytosolic E3 ubiquitin ligase. Activated Parkin ubiquitinates several OMM proteins, including VDAC1 and Mfn2, via a feed-forward amplification loop that promotes mitochondrial tagging. These ubiquitinated mitochondria are recognized by autophagic adaptors such as optineurin (OPTN), neural dot protein 52 kDa (NDP52), and p62, which fuse them to LC3-positive autophagosomal membranes [113,114,115]. Finally, the autophagosomes fuse with lysosomes for degradation, completing the mitophagy cycle.

Studies in post-mortem PD human brains has shown discrete populations of phosphorylated ubiquitin (p-Ub) structures within the mitochondrial clusters colocalized with the lysosome, indicating a blockage in autophagic flux [116]. These p-Ub positive structures accumulate in an age-and Braak stage- dependent manner, suggesting an age-dependent impairment of mitophagy with disease progression. Mutations in PINK1 or PARK2 (Parkin) impair mitochondrial turnover, leading to accumulation of dysfunctional mitochondria and augment ROS production and loss of dopaminergic neurons [117,118,119]. Thus, timely removal of damaged mitochondria via PINK1/Parkin-dependent mitophagy is critical for neuronal survival and represents a potential therapeutic intervention to curb inflammasome-mediated neurotoxicity.

#### 5.1.2. Parkin-Independent and Receptor-Mediated Mitophagy

**Figure 2:**
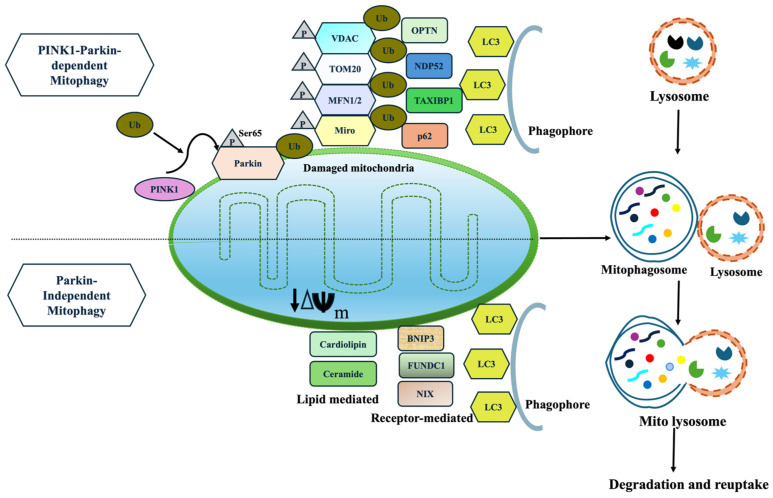
(i) Parkin-dependent mitophagy is initiated when mitochondria lose its membrane potential. PINK1 accumulates on the OMM, leading to phosphorylation (Ser65) and ubiquitination of Parkin, further recruiting and ubiquitinating widespread OMM proteins, including MFN1/2, VDAC, Tom20, and Miro. These OMM proteins are recognized by autophagy receptors (OPTN, NDP52), then link ubiquitinated mitochondria to LC3 and drive autophagosome formation and degradation. (ii) Parkin-independent mitophagy proceeds without Parkin through (a) receptor-mediated pathways (FUNDC1, AMBRA1, BNIP3/NIX) and (b) lipid-mediated signals (cardiolipin, ceramide). These inner and outer membrane proteins contain LC3-interacting region (LIR) motifs that allow direct binding to LC3 for degradation.

Beyond canonical Parkin-dependent mitophagy, receptor-mediated pathways provide alternative mechanisms for mitochondrial clearance. These pathways involve modulation of mitochondrial fission and fusion dynamics, primarily by inhibiting OPA1 and activation of Drp-1, facilitating mitochondrial fragmentation [120]. OMM proteins such as BNIP3, NIX, and FUNDC1 linked damaged mitochondria to autophagic machinery [121]. Under mitochondrial depolarization, USP19 and PGAM5 mediate FUNDC1 dephosphorylation, which enhances Drp1-dependent mitophagy [122]. Additional regulators such as AMBRA1 stabilize PINK1 and recruit alternative E3 ligases such as ARIH1, SIAH1, and HUWE1 to promote mitophagy [123]. Impaired mitochondrial fission or defective AMBRA1-mediated ubiquitination augment mitochondrial dysfunction, leading to uncontrolled oxidative stress, α-synuclein accumulation, and dopaminergic neuronal death [54]. Targeting these Parkin-independent pathways may therefore offer novel insights to restore mitochondrial homeostasis and slow neurodegeneration in PD (Figure 2).

**Figure 3:**
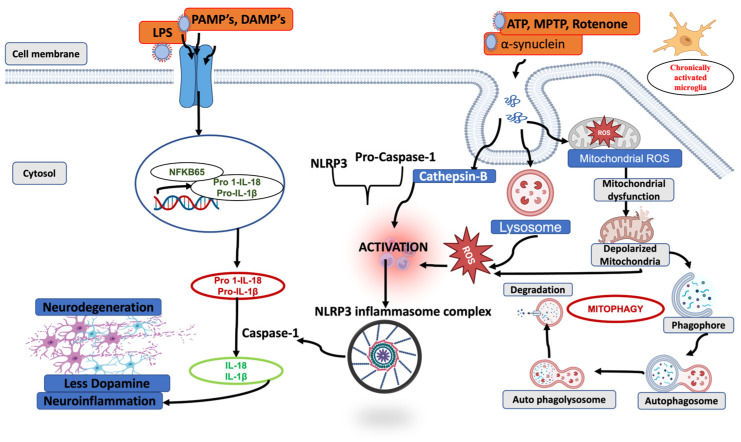
A schematic representation of the interplay between impaired mitophagy and NLRP3 inflammasome activation. The figure illustrates how defects in mitochondrial quality control led to the accumulation of damaged or depolarized mitochondria, resulting in excessive release of mitochondrial danger signals. These signals enhance NLRP3 inflammasome priming and activation, promoting ASC assembly and caspase-1 activation. Activated caspase-1 cleaves pro–IL-1β and pro–IL-18 into their mature forms, driving downstream neuroinflammation and contributing to progressive neurodegeneration.

Both Parkin-mediated and receptor-mediated mitophagy play essential roles in maintaining mitochondrial turnover, limiting ROS production, and preventing accumulation of harmful DAMPS (ATP, mtDNA α-synuclein). Impaired mitophagy results in excessive ROS generation, NLRP3 redistribution, and amplification of inflammatory signaling. This in turn aggravates mitochondrial damage and activates caspase-1 activity, along with the activation of IL-1β and IL-18, causing pyroptosis (Figure 3). Several pharmacological agents have been identified to mitigate neuroinflammation in PD by enhancing mitophagy and suppressing NLRP3 inflammasome activation. Neurotoxic prion peptide rP106-126 activates NLRP3 inflammasome and negatively regulates autophagy [124]. Andrographolide promotes Parkin-dependent mitophagy, effectively reducing NLRP3-mediated inflammation in both in vitro and MPTP-induced PD models [125]. Similar findings were shown where Urolithin promotes mitophagy and suppresses NLRP3 activity in LPS-induced BV2 microglia and the MPTP model of PD [126]. Cycloastragenol also reduces microglial NLRP3 activity in the PD model by promoting autophagy [127]. Pramipexole inhibited astrocytic NLRP3 inflammasome activation via Drd3-dependent autophagy in a mouse model of Parkinson’s disease [128]. Palmatine ameliorates dopaminergic neurodegeneration by regulating NLRP3 through mitophagy [129]. Collectively, these findings highlight autophagy/mitophagy-inducing compounds as promising therapeutic strategies for targeting neuroinflammation in PD.

### 5.2. Direct Degradation of Inflammasomes Through Autophagy

Compelling evidence links NLRP3 inflammasome overactivation in microglia to PD pathology across patient samples, animal models, and in vitro systems [107,130,131]. Under physiological conditions, microglia act as surveillance cells, clearing misfolded protein aggregates and damaged neurons while maintaining brain homeostasis. However, chronic microglial activation leads to excessive production of IL-6 and TNF-α, along with mature IL-1β and IL-18 through NLRP3 dependent pathway. Autophagy provides a critical break on this inflammatory cascade by directly degrading active inflammasome components. Studies demonstrate the co-localization of NLRP3 inflammasome components with the autophagic marker LC3, facilitating inflammasome clearance and limiting cytokine release. Qin et Al. have shown that a microglial-specific knockout of Atg5 enhances NLRP3 activation and accelerates dopaminergic neuron death in a MPTP model of PD [132]. Moreover, a p38–TFEB axis have introduced shown to inhibit chaperone-mediated autophagy (CMA)-dependent degradation of NLRP3 in α-synuclein A53T transgenic mice promoting inflammasome activation [133]. Thus, autophagic degradation of NLRP3 represents a plausible therapeutic strategy to interrupt early form of neuroinflammatory cascades in PD.

## 6. Drug Targets

### 6.1. Autophagy/Mitophagy Enhancers

Enhancing autophagy and mitophagy has emerged as a promising neuroprotective strategy in PD, aiming to mitigate α-synuclein aggregation, improve mitochondrial function, and reduce neuroinflammation. Several pharmacological agents have been identified that activate autophagic pathways, offering potential therapeutic avenues, as listed in Table 1. These compounds have consistently demonstrated that enhancement of autophagy/mitophagy improves neuronal survival, preserves mitochondrial integrity, and attenuates inflammatory responses in cellular and animal models of PD. However, most autophagy and mitophagy-inducing agents have not progressed beyond preclinical evaluation in PD due to poor blood–brain barrier penetration, dose-limiting toxicity, and pleiotropic systemic effects. Furthermore, the lack of PD-specific clinical trials and ongoing safety concerns underscore the need for next-generation autophagy/mitophagy modulators with improved specificity, enhanced brain penetrance, and controllable activity.

### 6.2. NLRP3 Inhibitors

Several NLRP3 inflammasome inhibitors have been identified that can effectively suppress neuroinflammation in PD by directly blocking NLRP3 activation, preventing its oligomerization, or inhibiting downstream cytokine production such as IL-1β, IL-18, and active Caspase-1. Some of them are summarized in Table 2, with their mechanistic mechanism. Preclinically, all these compounds consistently demonstrate strong inhibition of NLRP3 signaling, reduce microglial activation, attenuate pyroptosis, preserve nigrostriatal dopaminergic neurons, and improve motor outcomes. However, despite robust anti-inflammatory efficacy in vitro and in vivo, clinical translation remains limited. Only a small subset of NLRP3 inhibitors, such as MCC950 and dapansutrile (OLT1177), have advanced to clinical trials, and none have yet been approved for PD. Major obstacles include hepatotoxicity (e.g., MCC950), insufficient blood–brain barrier penetration, off-target immunosuppression, metabolic liabilities, and the absence of PD-specific clinical trial data for most compounds.

### 6.3. Dual Modulation

Studies have suggested that simultaneous targeting of mitophagy and NLRP3 inflammasome activation offers a beneficial outcome in the pre-clinical model of PD. Impaired mitophagy leads to the accumulation of damaged mitochondria, excessive ROS generation, and subsequent activation of the NLRP3 inflammasome. Therefore, pharmacological agents or interventions that enhance mitophagy while concurrently suppressing NLRP3 activation may synergistically reduce neuroinflammation. Some studies have demonstrated the additive or synergistic neuroprotective effects of such dual modulators are listed below (Table 3). Overall, these dual-modulator strategies exemplify how targeting both upstream triggers (damaged mitochondria) and downstream effectors (inflammasome activation) can achieve synergistic neuroprotection, highlighting the therapeutic potential of integrated approaches in PD.

### 6.4. Drugs in Clinical Trials

Below is a summary of some clinical-trial agents that either modulate NLRP3 inflammasomes’ activity or enhance autophagy/mitophagy in PD, as listed in Table 4. Several compounds have advanced into early-phase clinical trials, reflecting growing translational interest in modulating these mechanisms to slow disease progression. However, challenges remain, including defining optimal patient stratification, treatment timing, long-term safety, and reliable biomarkers of target engagement and disease modification.

## 7. Limitations of Current Therapeutic Strategies

Despite significant advances achieved in understanding the molecular mechanisms underlying PD, several limitations remain in translating NLRP3 inflammasome inhibitors and autophagy/mitophagy modulators into effective clinical therapies. Although several IL-1β–targeted therapies, such as anakinra, canakinumab, and rilonacept, have shown efficacy in various neuroinflammatory diseases, no clinical trials in PD or other neurodegenerative disorders have been reported, mainly due to poor blood–brain barrier (BBB) permeability and limited therapeutic efficacy [168]. Moreover, since IL-1β can also be produced via inflammasome-independent pathways, blocking it directly may lead to undesired immunosuppression [25].

Other NLRP3 inhibitors, such as MCC950, β-hydroxybutyrate, Bay 11-7082, and Ac-YVAD-CMK, have shown promising anti-inflammatory effects in vitro and in pre-clinical PD models. However, these compounds remain largely off target, exhibit low potency, poor BBB permeability, and short biological half-lives and lack human trail data [169,170]. In addition, inhibitors such as MCC950 and β-hydroxybutyrate have serious long-term adverse effects, including hepatotoxicity, metabolic disturbances and off-target immune modulation, which significantly hinder their translational potential [171]. Another drawback of these NLRP3 inhibitors is that prolonged suppression of NLRP3 activity may impair physiological immune responses, thereby increasing vulnerability to infections and diminishing normal tissue repair processes, which highlights an important long-term safety concern [172]. Additionally, IL-18 and other pro-inflammatory mediators involved in PD pathology still lack specific inhibitors [173]. Collectively, these limitations raise safety and translational concern prompting an urgent need for novel therapeutics targeting upstream regulators of NLRP3 activation that can effectively cross the BBB and modulate neuroinflammation in PD.

Similarly, enhancing mitophagy through pharmacological agents such as PINK1/Parkin activators (e.g., spermidine, urolithin A), mTOR inhibitors (rapamycin), NAD^+^ precursors, and AMPK/SIRT1 activators has demonstrated neuroprotective effect in preclinical PD models, including improved mitochondrial quality control, reduced neuroinflammation, and preservation of dopaminergic neurons [113,174,175]. However, several unresolved challenges remain. Both excessive and insufficient mitophagy can be detrimental, making its regulation a double-edged sword for neuronal survival [104,176]. Although many studies advocate the benefits of mitophagy enhancement, increasing mitophagic flux is not always therapeutically beneficial and may exacerbate mitochondrial stress under certain pathological conditions [176]. Therefore, mitophagy offers therapeutic benefits; however, precise modulation, rather than indiscriminate activation, is essential to achieve neuroprotection.

Furthermore, a more in-depth mechanism of mitophagy must be elucidated, showing that its interconnection with different mitophagy pathways is required. Untangling the multifaceted roles of mitophagy in neuronal survival and death is needed, and mitophagy can be both beneficial and detrimental for the neuronal bioenergetics depending on the pathological condition. Since mitochondria are highly dynamic, the precise stage of mitochondrial morphology (fusion and fission) must be determined to develop mitophagy inducers [177]. Moreover, the development of specific, potent, and low-toxicity mitophagy inducers with demonstrable clinical benefits is still a missing linkage [178,179]. The dual role of mitophagy in promoting both cell survival and death, together with the poor specificity and safety profile of current inducers and lack of reliable in vivo markers to monitor mitophagy, further complicate the current therapy [180,181].

## 8. Conclusions

Several studies have highlighted the critical role of NLRP3 inflammasome activation and defective mitophagy as key drivers for neuroinflammation and dopaminergic neurodegeneration in PD. Dysregulated mitochondrial quality control promotes the accumulation of damaged mitochondria and releases DAMP’s, which, in turn, amplify NLRP3 inflammasome signaling and sustain neuroinflammatory cascades. Consequently, targeting the inflammasome–mitophagy axis, either individually or in combination, emerges as a compelling therapeutic strategy for slowing PD progression.

Despite promising preclinical outcomes, the clinical translation aimed to target NLRP3 inflammasome activity and autophagy/mitophagy in PD remains highly challenging. Major barriers include an incomplete understanding of the underlying molecular mechanisms, poor blood–brain barrier (BBB) permeability of current agents, limited target specificity, off-target toxicities, and inconclusive clinical outcomes. Moreover, indiscriminate inhibition of NLRP3 or excessive mitophagy may disrupt physiological immune responses and mitochondrial homeostasis, underscoring the need for precise and context-dependent modulation. These challenges highlight the need for more refined and targeted therapeutic strategies to safely harness the neuroprotective potential of inflammasome–mitophagy regulation in PD.

## 9. Future Perspectives

Future research should prioritize elucidating the precise molecular mechanisms that link mitophagy dysfunction to NLRP3 inflammasome activation in PD models. A deeper understanding of upstream regulatory mechanisms—such as post-translational modifications and specific phosphorylation sites governing NLRP3 activation—may enable the development of highly specific therapeutic interventions with reduced off-target effects [182,183]. In parallel, structure–activity relationship (SAR)-based optimization, comprehensive pharmacokinetic profiling, and rigorously designed clinical trials will be essential to establish the safety and efficacy of inflammasome- and mitophagy-targeted therapies.

A critical gap lies in the lack of robust, clinically translatable biomarkers capable of monitoring mitophagy and inflammasome activity in vivo. Conventional techniques such as [18F]-FDG PET and fMRI primarily assess glucose metabolism and functional connectivity, offering only indirect and non-specific information about mitophagy and inflammasome activity [184]. Instead, newer radiopharmaceuticals techniques, such as second-generation TSPO ligand, could provide a direct way to trace activated microglia and neuroinflammatory process in vivo. However, their interpretation is challenging, because TSPO expression is not specific to the microglial phenotype, and cellular heterogeneity impairs signal outcome. Another major gap that needs to be addressed is the disconnect between molecular mechanisms and clinically measurable parameters. For example, circulating cell-free mtDNA may reflect impaired mitophagy, but its relationship to neuronal mitophagy remains unsolved. Bridging this gap will require the development of integrated biomarker panels that combine cytokine profiling, circulating cell-free mtDNA, TSPO-based PET imaging, and next-generation mitochondrial PET tracers.

Advanced techniques, including phosphorus magnetic resonance spectroscopy and mitochondrial-PET tracers, may allow real-time visualization and assessment of mitochondrial dynamics without triggering excessive mitophagy or energy depletion [185]. Additionally, the combination of therapeutic strategies that simultaneously inhibit NLRP3 inflammasome activation, promote controlled mitophagy, and exhibit better BBB-penetrating ability may provide superior potential by targeting both upstream pathogenic triggers and downstream inflammatory cascades [186]. Ultimately, integrating mechanistic insights with innovative therapeutic and biomarker development will be crucial for translating inflammasome mitophagy-based strategies into effective clinical interventions.

## Figures and Tables

**Table 1:** Summary of pharmacological agents that enhance autophagy, detailing their mechanistic evidence (in vitro and in vivo), stage of development, and key limitations relevant to PD. Arrows indicate direction of effects: ↑, increase; ↓, decrease; →, mechanistic linkage.

Compound	Mechanistic Evidence (from In Vitro/In Vivo Studies)	Developmental Stage	Major Limitation/Cause of Failure	Reference
Rapamycin (Sirolimus)	In vitro: Primary neurons. Rapamycin increases autophagic flux by enhancing autophagosome formation and autophagosome–lysosome fusion, evidenced by LC3-II turnover. In vivo: Not reported in this study.	Preclinical (PD models)	Immunosuppressant; metabolic side effect; narrow therapeutic window; poor BBB penetration	[134]
Metformin	In vitro (adult DRG sensory neurons): Metformin activates AMPK; suppresses mitochondrial electron transport; promotes neurite outgrowth; proposed to upregulate autophagy via AMPK → likely mTORC1 suppression. In vivo (sciatic nerve injury model): Metformin increases LC3-II (autophagy marker), enhances autophagy; correlates with reduced cell death, improved myelination and motor recovery. Autophagy inhibition abolishes these benefits.	Preclinical (PD-related models); epidemiological studies	Conflicting clinical outcome; metabolic side effect	[135]
Trehalose	NSC-34 and primary motoneurons. Trehalose activates TFEB via lysosomal mechanisms, increasing autophagy and lysosomal biogenesis; reduces misfolded protein accumulation. trehalose induces low-grade lysosomal stress → TFEB activation → enhanced autophagy–lysosome gene expression and lysosomal expansion. In vivo: In mice, trehalose induces TFEB activation in liver tissue, increasing autophagy–lysosome biogenesis.	Preclinical	Poor bioavailability; rapid degradation by trehalase; limited BBB penetration	[136,137]
Spermidine	In vitro: primary neuron-spermidine induces autophagy by preventing caspase-3–mediated cleavage of Beclin-1, preserving autophagy initiation; increases autophagic flux and protects against apoptosis. Summarizes evidence that spermidine activates autophagy through an mTOR-independent pathway in various in vitro models (cell types not specified in the review). In vivo: Spermidine enhances neuronal autophagy, reduces caspase-3 activity, preserves Beclin-1, and provides neuroprotection. Summarizes that spermidine has autophagy-dependent neuroprotective and anti-aging effects in multiple in vivo models.	Preclinical	Dose optimization unclear; long-term safety in CNS not established	[138,139]
Resveratrol	BMDMs and THP-1 cells. Resveratrol activates autophagy, preserves mitochondrial integrity, and inhibits NLRP3 inflammasome (↓ caspase-1 cleavage, ↓ IL-1β); autophagy is required for this effect. Summarizes that resveratrol activates SIRT1/AMPK → autophagy and inhibits NLRP3 inflammasome in immune cells. In vivo: Mouse model. Resveratrol increases autophagy in splenocytes and reduces NLRP3 inflammasome activation; confirms autophagy-dependent anti-inflammatory effects. Summarizes in vivo data from prior studies supporting autophagy-dependent inhibition of NLRP3 by resveratrol.	Preclinical; limited clinical studies (non-PD)	Poor stability and bioavailability; low BBB penetration	[140,141]

**Table 2:** Summary of pharmacological agents targeting NLRP3 inflammasome activation, detailing mechanistic evidence (in vitro and in vivo studies), stage of development, and key limitations relevant to PD. The arrow (→) denotes downstream signaling or mechanistic linkage.

Compound	Mechanistic Evidence (In Vitro/In Vivo)	Developmental Stage	Major Limitation/Cause of Failure	Reference
MCC950	In vitro: Selectively inhibits NLRP3 by blocking NACHT ATPase activity → prevents NLRP3 oligomerization, ASC speck formation, and IL-1β/IL-18 release in macrophages; no effect on AIM2 or NLRC4 inflammasomes. In vivo: Reduces IL-1β levels and inflammation in NLRP3-driven mouse models (LPS septic shock, MWS/CAPS models, EAE), improving survival and clinical symptoms via selective NLRP3 inhibition.	Terminated in Phase II trial	Hepatotoxicity; long-term safety concern	[142]
Dapansutrile	In vivo: In EAE mice, it reduces IL-1β, IL-18, IL-6, and TNFα in spinal cord; attenuates immune cell infiltration and demyelination, improving clinical scores, consistent with selective NLRP3 inflammasome inhibition. In vitro: Not reported.	Phase II/III trail	Limited CNS efficacy data	[143]
β-Hydroxybutyrate (BHB)	In vitro (macrophages): BHB selectively inhibits NLRP3 by blocking K^+^ efflux, preventing ASC oligomerization, caspase-1 activation, and IL-1β /IL-18 production. In vivo (mouse models): BHB suppresses NLRP3-driven inflammation in gout, LPS-induced inflammation, and MWS models, reducing IL-1β levels and neutrophil recruitment.	Pre-clinical	Limited specificity; BBB penetration unclear	[144]
Curcumin	In vitro (neuronal and inflammatory cell models): Curcumin inhibits the HDAC6–NLRP3 pathway, reducing NLRP3 activation and inflammatory signaling. In vivo (PD mouse model): Curcumin suppresses HDAC6-dependent NLRP3 activation, decreases neuroinflammation, and protects dopaminergic neurons.	Pre-clinical	Poor bioavailability; limited BBB penetration	[144,145]
Minocyclin	Suppresses microglial activation; inhibits NF-κB priming and NLRP3 activation. Reduces neuroinflammation and dopaminergic neuron loss in MPTP and 6-OHDA models.	Pre-clinical	Long-term toxicity concern	[146]
Salidroside	In vitro (PC-12/BV2 cells): Salidroside inhibits NLRP3-dependent pyroptosis by suppressing TLR4/NF-κB and TXNIP/NLRP3/caspase-1 signaling. In vivo (MPTP-PD mice): Reduces NLRP3 activation, IL-1β/IL-18, and GSDMD cleavage; protects dopaminergic neurons and improves PD symptoms.	Pre-clinical	No clinical trial data available; Pharmacokinetics unclear	[147]
Usnoflast (ZYIL1)	In vitro: ZYIL1 inhibits NLRP3 activation and IL-1β release in THP-1 cells, PBMCs, and microglia. In vivo: Reduces NLRP3 activation and IL-1β, protects dopaminergic neurons, and improves motor deficits in PD mouse models.	Pre-clinical	No clinical data; long-term safety data unknown	[148]
Baicalein	In vitro (glial cells): Baicalein inhibits NLRP3, caspase-1, IL-1β, and GSDMD cleavage, reducing pyroptosis. In vivo (MPTP-PD mice): Suppresses NLRP3/caspase-1/GSDMD pathway, decreases inflammation, protects dopaminergic neurons, and improves motor function.	Pre-clinical	Limited specificity; lack of clinical validation	[149]
Echinacoside	In vivo (MPTP-PD mice): Echinacoside inhibits NLRP3/caspase-1/IL-1β signaling, protects dopaminergic neurons, and improves motor behavior. In vitro: Not reported.	Pre-clinical	No invitro mechanistic data available	[150]
Tubastatin A	In vitro (SH-SY5Y cells): Tubastatin A inhibits NLRP3, caspase-1, and IL-1β activation. In vivo (6-OHDA PD mice): Reduces NLRP3-mediated inflammation, protects dopaminergic neurons, and improves nigrostriatal integrity.	Pre-clinical	Lack of safety concern; off-target effect seen	[151]
Dl-3-n-Butylphthalide	In vitro/In vivo: This study does not report experiments or mechanistic data for DL-3-n-Butylphthalide. The referenced paper examines Tubastatin A (HDAC6 inhibition) rather than NBP.	Not validated	No direct NLRP3 mechanistic evidence	[151]
Glibenclamide	In vitro (BV2 cells): Inhibits NLRP3, caspase-1, and IL-1β. In vivo (paraquat + PD mice): Reduces NLRP3 activation, protects dopaminergic neurons, and improves motor function.	Pre-clinical	Systemic hypoglycemia risk; off target effects	[152]
Cyclosporine A	In vitro (HT22 cells): NIM811 (CsA derivative) inhibits NLRP3, caspase-1, GSDMD, IL-1β/IL-18, and pyroptosis. In vivo: Not reported.	Pre-clinical	Immunosuppression; no in vivo PD validation	[153]
KPT-8602	In vitro (BV2 cells): Inhibits NF-κB, NLRP3, caspase-1, and IL-1β. In vivo (PD mice): Suppresses NF-κB/NLRP3 signaling, reduces neuroinflammation, and protects dopaminergic neurons.	Pre-clinical	No clinical data	[154]
Cordycepin	In vitro (BV2 cells): Inhibits TLR4/NF-κB, NLRP3, caspase-1, and IL-1β. In vivo (LPS-PD mice): Suppresses TLR4/NF-κB/NLRP3 signaling and protects dopaminergic neurons.	Pre-clinical	Bioavailability and safety concern	[155]
CY-09	In vitro/in vivo: No direct data for CY-09; the study focuses on p38-TFEB regulation of NLRP3 in microglia.	Mechanistic data only	No direct NLRP3 inhibition data	[133]
Oridonin	In vitro: Blocks NEK7–NLRP3 interaction, preventing caspase-1 activation and IL-1β/IL-18 release in macrophages. In vivo: Reduces NLRP3-mediated inflammation in mouse models of peritonitis, gout, and type 2 diabetes.	Pre-clinical	Toxicity concern; no PD-specific studies	[156]

**Table 3:** Summary of dual modulators targeting autophagy/mitophagy and NLRP3 inflammasome pathways, detailing its mechanistic rationale and experimental evidence (in vitro and in vivo studies). The arrow (→) denotes downstream signaling or mechanistic linkage.

Combination	Mechanistic Rationale	Mechanistic Evidence (In Vitro/In Vivo)	References
Rapamycin + MCC950	Activates autophagy (Rapamycin) and inhibits NLRP3 inflammasome (MCC950) → synergistic neuroprotection.	In vitro (cortical neurons): Rapamycin activates autophagy; MCC950 inhibits NLRP3, reducing caspase-1 and IL-1β/IL-18. In vivo (TBI mice): Combination enhances neuroprotection, suppresses NLRP3 activation, and reduces neuronal damage.	[157]
Metformin + Resveratrol	Activates AMPK/SIRT1 → promotes autophagy and suppresses NLRP3 simultaneously.	In vitro (3T3-L1 cells): Activates AMPK, inhibits Drp1-mediated mitochondrial fission, ER stress, and NLRP3. In vivo (diabetic mice): Increases AMPK, reduces ROS, mitochondrial fission, ER stress, and NLRP3 activation.	[158]
Trehalose + β-Hydroxybutyrate	Trehalose promotes TFEB-mediated lysosomal/autophagy function; BHB inhibits NLRP3 → combined clearance and anti-inflammatory effect.	In vitro: BHB blocks NLRP3 activation in macrophages. In vivo: Reduces NLRP3-dependent IL-1β and inflammation in mice.	[144]
Kaempferol	Inhibit NLRP3 inflammasome activation and promote autophagy.	In vitro: Inhibits NLRP3 and IL-1β; promotes autophagy in BV2 cells. In vivo: Enhances autophagy, suppresses NLRP3, and protects dopaminergic neurons.	[146]
Andrographolide	Inhibit NLRP3 inflammasome activation and promote mitophagy.	In vitro: Induces parkin-mediated mitophagy; inhibits NLRP3 and IL-1β in microglia. In vivo: Enhances mitophagy, suppresses NLRP3, and protects dopaminergic neurons.	[125]
Perillyl Alcohol	Inhibit NLRP3 inflammasome activation by scavenging ROS production.	In vitro (microglia): Scavenges ROS, inhibits NLRP3 activation, and reduces IL-1β release. In vivo: Reduces ROS and NLRP3 inflammasome activation, protecting dopaminergic neurons.	[159]

**Table 4:** Summary of compounds targeting NLRP3 inflammasome or autophagy/mitophagy pathways in PD detailing developmental stage and its key outcomes.

Compound	Targets	Developmental Stage	Randomized	Treatment Duration	Therapeutic Potential, Safety, Efficacy	Outcomes/Key Finding	References/Patents/Trial
NT-0796 (Nod Thera)	NLRP3 inflammasome inhibitor	Phase Ib/2a trial completed	No	28 days, with measurable effects as early as 7 days	Selective, brain-penetrant NLRP3 inhibitor showing anti-neuroinflammatory activity in PD; potential immunosuppression risk.	Safe, target engagement, biomarker reduction (NfL, sTREM2 in CSF); No direct data on α-synuclein. Next trail planned.	[160]
VTX3232-(Ventyx biosciences)	NLRP3 inhibitor	Phase 2a	No	28 days daily oral dosing (40 mg)	Brain-penetrant, orally available NLRP3 inhibitor modulating microglial-driven neuroinflammation potential immune-related risks, long-term CNS safety untested.	Safe, reduced inflammation. No direct data on α-synuclein. Not failed.	[161], NCT06556173
Dapansutrile (Olatec) (OLT1177)	NLRP3 inhibitor	Phase II “DAPA-PD”	Not yet conducted	Design details unpublished as of 2025	Oral NLRP3 inhibitor modulating microglial activity and reducing α-synuclein neurodegeneration; well-tolerated with high safety, suitable for early or inflammation-driven PD.	Preclinical work suggests possible relevance, but no clinical PD data.	[162]
ISM8969	N/A	Complete IND-enabling studies	N/A	N/A	Preclinical compound with completed IND-enabling studies; mechanism not disclosed. Safety, efficacy, and clinical potential remain untested, positioned for early-stage development.	Motor benefits in mice. Positive α-synuclein effects preclinically.	[163]
Metformin	AMPK activator, improve mitophagy	No PD RCTs’ observational only	Years (observational)	N/A	AMPK activator enhancing mitophagy in humans; well-tolerated, limited PD-modifying effect, potential adjunct or preventive therapy.	Mixed findings, no clear protective benefits, Preclinically have some effect on α-synuclein.	[164]
Rapamycin (Sirolimus)	mTOR inhibitor	Pre-clinical complete/No PD trials	N/A	N/A	mTOR inhibitor inducing autophagy with preclinical neuroprotective effects in PD models; significant immunosuppression limits long-term use, high disease-modifying potential but translational risk remains.	Preclinical data showed neuroprotective.	[165]
Urolithin A	Mitophagy inducer	N/A	N/A	N/A	Mitophagy inducer that promotes mitochondrial quality control; well-tolerated with minimal safety concerns, modest disease-modifying potential, suitable for preventive or adjunctive use in PD	No data clinically (Theoretical).	[166]
MTX325 (Mission Therapeutics)	USP30 inhibitor (mitophagy enhancer)	Phase I	N/A	N/A	USP30 inhibitor enhancing mitophagy and protecting dopaminergic neurons in PD models; early-phase clinical testing, safety and efficacy in humans not yet established, promising disease-modifying potential.	N/A	[167]
ABBV-1088	PINK1 activator	Phase I (NCT06414798/NCT06579300)	Early phase data	No-placebo-controlled PD efficacy trials reported as of now (2025)	PINK1 activator targeting mitophagy in neurodegenerative disease.	Phase 1 results published mainly pharmacokinetics/safety in healthy subjects	[37], WO2021168446A1
VNA-318	Mitophagy activator	Phase I (NCT06721091)	Early trial	No-placebo-controlled PD efficacy trials reported as of now (2025)	Mitophagy activator in Phase I trials; safety and efficacy in humans untested, potential disease-modifying agent for PD pending clinical validation.	N/A	[37], US20220105117A1
Selnoflast/Inflazome (Roche)	NLRP3 inflammasome inhibitor	Phase 1b with safety and tolerability in patients with early idiopathic PD	N/A	Not yet randomized	Brain-penetrant NLRP3 inhibitor showing safety and tolerability in early idiopathic PD; potential immune-related risks exist. Comparable efficacy to other NLRP3 inhibitors, with promise for early or inflammation-driven PD.	Data has not been released	[161]

## Data Availability

The original contributions presented in this study are included in the article. Further inquiries can be directed to the corresponding author.

## Abbreviations

The following abbreviations are used in this manuscript:

PD	Parkinson’s Disease
SNpc	Substantia Nigra Par Compacta
CNS	Central Nervous System
HLA-DR	Human Leukocyte Antigen-D-Related
DAMPS	Damage-Associated Molecular Patterns
ROS	Reactive Oxygen Species
IL-1β	Interleukin-1 beta)
Il-18	Interleukin-18
NLRP3	NOD-, LRR-, and Pyrin Domain-Containing Protein 3
PAMPs	Pathogen-Associated Molecular Patterns
MPTP	1-methyl-4-phenyl-1, 2, 3, 6-tetrahydropyridine
PYHIN/HIN	Hematopoietic Interferon-Inducible Nuclear
AIM2	Absent in Melanoma 2
IFI16	Interferon-Gamma Inducible Protein 16
ATP	Adenosine Triphosphate
NACHT	Nucleotide-Binding
LRR	Leucine Rich Repeats
CARD	Caspase Recruitment Domain
TLR	Toll-like Receptor
NF-κB	Nuclear Factor Kappa-Light-Chain-Enhancer of Activated B Cells
TNF	Tumor Necrosis Factor
CSF	Cerebrospinal Fluid
AD	Alzheimer’s Disease
SNP	Single-Nucleotide Polymorphisms
PARP	Poly (ADP-ribose) Polymerase 1
iPSC	Induced Pluripotent Stem Cell
mtDNA	Mitochondrial DNA
CMA	Chaperone-Mediated Autophagy
LAMP2A	Lysosomal-Associated Membrane Protein 2
ULK1	Unc-51-Like Autophagy Activating Kinase 1
mTORC1	Mechanistic Target of Rapamycin Complex 1
LC3I	Microtubule-associated protein 1 light chain 3
ATG	Autophagy-Related
SQSTM1	Sequestosome 1
PINK1	PTEN-Induced Putative Kinase 1
OMM	Outer Mitochondrial Membrane
OPTN	Optineurin
FUNDC1	FUN14 Domain Containing 1
OPA1	Optic Atrophy 1
BNIP3	Bcl-2/Adenovirus E1B 19-kDa-Interacting Protein 3
LPS	Lipopolysaccharide
AMPK	AMP-Activated Protein Kinase
fMRI	Functional Magnetic Resonance Imaging
PET	Positron Emission Tomography
TSPO	Translocator Protein
